# Self-organizing ontology of biochemically relevant small molecules

**DOI:** 10.1186/1471-2105-13-3

**Published:** 2012-01-06

**Authors:** Leonid L Chepelev, Janna Hastings, Marcus Ennis, Christoph Steinbeck, Michel Dumontier

**Affiliations:** 1 Department of Biology, Carleton University, Ottawa, Canada; 2 European Bioinformatics Institute, Wellcome Trust Genome Centre, Hinxton, UK; 3 School of Computer Science, Carleton University, Ottawa, Canada; 4 Institute of Biochemistry, Carleton University, Ottawa, Canada

## Abstract

**Background:**

The advent of high-throughput experimentation in biochemistry has led to the generation of vast amounts of chemical data, necessitating the development of novel analysis, characterization, and cataloguing techniques and tools. Recently, a movement to publically release such data has advanced biochemical structure-activity relationship research, while providing new challenges, the biggest being the curation, annotation, and classification of this information to facilitate useful biochemical pattern analysis. Unfortunately, the human resources currently employed by the organizations supporting these efforts (e.g. ChEBI) are expanding linearly, while new useful scientific information is being released in a seemingly exponential fashion. Compounding this, currently existing chemical classification and annotation systems are not amenable to automated classification, formal and transparent chemical class definition axiomatization, facile class redefinition, or novel class integration, thus further limiting chemical ontology growth by necessitating human involvement in curation. Clearly, there is a need for the automation of this process, especially for novel chemical entities of biological interest.

**Results:**

To address this, we present a formal framework based on Semantic Web technologies for the automatic design of chemical ontology which can be used for automated classification of novel entities. We demonstrate the automatic self-assembly of a structure-based chemical ontology based on 60 MeSH and 40 ChEBI chemical classes. This ontology is then used to classify 200 compounds with an accuracy of 92.7%. We extend these structure-based classes with molecular feature information and demonstrate the utility of our framework for classification of functionally relevant chemicals. Finally, we discuss an iterative approach that we envision for future biochemical ontology development.

**Conclusions:**

We conclude that the proposed methodology can ease the burden of chemical data annotators and dramatically increase their productivity. We anticipate that the use of formal logic in our proposed framework will make chemical classification criteria more transparent to humans and machines alike and will thus facilitate predictive and integrative bioactivity model development.

## Background

Over the hundreds of years of biochemical research, humanity has encountered myriads of chemical entities with countless combinations of functional groups that imparted upon their bearers distinct reactivities and properties. According to the structure-activity relationship (SAR) principle, grouping these entities into structure- and property-based classes within a larger chemical ontology (formal logical specification of a chemical hierarchy conceptualization) can enable the recapitulation of or improvements in the prediction of their biological functionality and chemical reactivity patterns, thus providing indispensable assistance in understanding the molecular nature of metabolism, toxicity, and bioactivity [1,2]. In addition to this, the assignment of individual chemical entities to a given class within a chemical ontology or hierarchy may facilitate the inference of the potential anticipated roles and properties of these entities. This capacity of chemical ontologies may help support the development of the rising systems sciences, such as chemogenomics and systems chemistry [3].

Despite the numerous efforts to develop automated chemical classification and ontology construction approaches (discussed below), this process has so far practically remained firmly within the hands of human curators, as exemplified by perhaps the largest chemical ontology constructed to date - the Chemical Entities of Biological Interest (ChEBI) database and ontology [4]. As more chemical data becomes readily available from large-scale experimentation, ChEBI and numerous similar repositories of biological and chemical information (e.g. [5-7]) are increasingly coming under strain due to lack of human curators, the overwhelming wealth and diversity of the new chemical information, and the boundlessness of the chemical space. Clearly, a means to automate and facilitate these classification efforts is urgently needed if we desire to understand, expose, and integrate the entirety of available chemical information.

Manual chemical classification efforts frequently involve the tedious work of multiple human experts who carefully peruse the relevant chemical literature to gain competence in assigning chemical entities to a range of classes. Although the reliance of biochemical activity-based classification efforts on the predictions or informed estimates of human experts has been largely relegated to the informal or speculative domain of scientific communications thanks to the development of numerous quantitative structure-activity relationship (QSAR) elucidation approaches [8], chemical hierarchy construction is still very much a human-dominated domain. Chemical hierarchies in common use, such as ChEBI and the Medical Subject Headings (MeSH) organic chemistry hierarchy [9], are structure-based but often have the capacity to incorporate chemical classes that are based on functional chemical attributes, such as those bearing a certain pharmaceutical activity (e.g. ChEBI antibiotics). Although structure- and function-based classification schemes involve somewhat different approaches for construction, the basic accepted SAR dogma is that chemical structure and chemical function are inseparably linked, as noted earlier. Therefore, structure-based classification efforts could be useful in initiating the construction of predictive models of small molecule bioactivity.

The correlation and overlap between chemical topology-based and biological function-based chemical hierarchies is expected since much of biological activity is enacted through interactions and transformations that are specific to a particular set of structural features of small molecules. For instance, most enzymes operate on a very limited set of chemical structures: carbonyl reductases, operate on carbonyl and alcohol functional groups while bacterial transpeptidases bind to and operate upon structures that resemble the peptide bond, leading to the potency of beta lactam-based drugs in the inhibition of their operation. While there may be more than one 2D chemical structure that results in an interaction with a given enzyme (due to its 3D configuration), we claim that there is a finite set of 2D chemical skeletons that result in a bioactive interaction, and that given a sufficiently large dataset of compounds known to be bioactive, it is possible to characterize a class of functionally active compounds as a collection of classes with well-defined, consistent structural features. Concerns for the predictive capacity of such classifications, like the distinction between stereoisomers and a range of chemical properties such as molecular volume that may result in disqualifying an otherwise well-suited 2D skeleton from a bioactivity-inducing interaction can be laid to rest by explicitly incorporating these features into class definition. In this work, we demonstrate that given the logical expressivity afforded by the Web Ontology Language (OWL) [10], we can automatically create formal logical definitions for a range of topology and property-based chemical classes which can then self-assemble into a chemical ontology.

While manual literature searching and experimental data analysis is often involved in the classification of chemical entities into functional classes, structure-based hierarchy construction is much more well-defined and easily amenable to automation. In constructing structure-based chemical classes, a modern curator would familiarize themselves with any existing informal class definitions, as well as any known chemical class members. Based on this expertise, the curator would then define a class, create a short textual or pictorial description of this class, and add child/parent relationships to the other classes, as well as including several representative entities. In essence, this approach requires the resulting ontology to consist of a formalisation of expert domain knowledge in the area, while providing no means for this knowledge to be captured in a machine-understandable format. The result of this is a lack of facile re-use of the work of human curators in classification and annotation of novel entities and establishing child/parent relationships for newly added classes when the ontology grows or is modified. Unfortunately, the majority of chemical ontologies, such as ChEBI database and ontology, MeSH chemical classifications, and LIPIDMAPS [11], suffer from this, resulting in artificial discipline-specific barriers to research, since none of these can be easily or automatically integrated. As the chemical ontologies grow in size and the range of relationships covered, manual ontology development becomes increasingly difficult due to the multiplying number of consistency checks that have to be performed for each new class and relationship added.

To date, automated chemical classification has mostly belonged to the domain of QSAR studies which involve the characterization of a training set of chemical entities with respect to a particular set of computable or measurable molecular features, as well as the property of interest, such as biochemical activity or biomedical potency [8]. The features that are used for training are often selected such that the cost of their acquisition is significantly lower than that of the modelled property. This feature set is then used to construct a predictive model using one of many available mathematical procedures, including the creation of decision trees [12], multidimensional parametric fits [13], or three-dimensional pharmacophore models [14], to name a few. The resultant models are then verified for their capacity to adequately classify molecules into a range of classes of interest using a test set of compounds not used in model creation. While the resultant models may often achieve a great degree of accuracy, the vast majority of such models are not readily amenable to human understanding or automated incorporation into a chemical hierarchy. In order to fully appreciate the structural and physicochemical molecular features of relevance to a particular functional or structural classification, post-processing of the resultant mathematical models is often required. Although we have recently demonstrated the conversion of decision tree-based QSAR models into formally axiomatized OWL chemical ontologies [15], the problem of integration of disparate, problem-specific functional chemical classifications into a single chemical ontology is still largely unsolved. Compounding this problem is the fact that chemical class definitions derived using statistical machine learning approaches are often inherently probabilistic - that is, no class membership requirement is formally stated as a necessary or sufficient condition. In order to derive a truly self-organizing chemical ontology, a deterministic classification in terms of well-defined features and their combinations is necessary.

While function-based chemical hierarchy construction has not been adequately addressed, methods to generate structure-based chemical ontologies have been studied in the past to some extent. For example in [16], a very early attempt at formalizing chemical classes and their relationships for chemical inference has been carried out, but never practically implemented for any large-scale classification exercise. In [17], a chemical ontology has been manually constructed with classes formally defined as containing molecules that possessed a particular functional group or a set of functional groups. While this work has been successfully applied for practical chemical functionality analysis through chemical semantic similarity identification enrichment, the basic ontology construction still followed a manual process and was limited to a set of pre-defined functional groups, insufficient for characterizing a number of more complex chemical classes. Furthermore, the lack of a formal, accessible framework for chemical classification (in the form of OWL) has resulted in limited logical chemical class axiom expressivity, therefore limiting the applicability of the approach for characterizing some chemical classes (e.g. those requiring cardinality restrictions, such as dienes). Finally, the work of [18] has resulted in a structure-based chemical ontology that employed combinations of chemical substructures of increasing complexity to produce increasingly specialized chemical classes, with the aim of improving the structural analysis and pattern identification in sets of biologically active compounds.

Unfortunately, a framework or approach for fully automated construction of chemical ontologies has never been achieved. In this work, we propose a radically novel approach for structure-based chemical classification automation by abstracting and automating the majority of chemical curation to construct machine- and human-understandable, formally axiomatized chemical class definitions in OWL. Further, we use these automatically generated chemical classes to enable machine reasoning over the ontology and demonstrate ontology self-organization, as well as classification of individual chemical entities represented using the Chemical Entity Semantic Specification formalism [19]. Finally, we demonstrate that certain functionally-relevant classes can be defined unambiguously using our approach, and integrated into the overall chemical ontology seamlessly.

## Results

**Figure 1:**
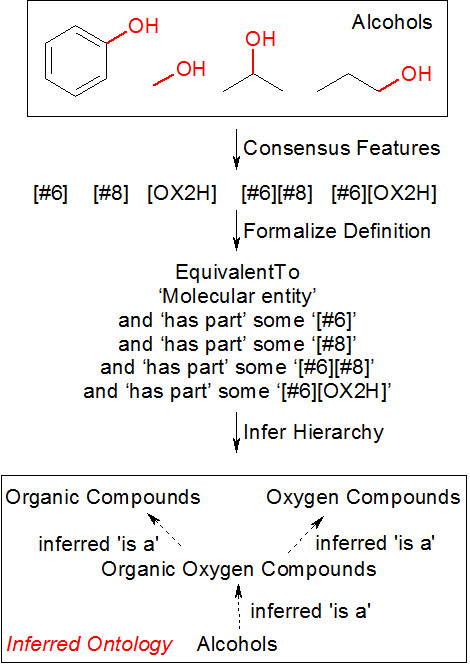
**An abstract view of the curation effort for structure-based chemical hierarchies**.

We have reduced the activity of a human curator to several steps, namely a) the identification of high-confidence chemical class members, b) identification of consensus chemical patterns that have to be present in a given compound to qualify for membership in a given class, c) creation of formal chemical class definition axioms, and d) chemical classification and class relationship assignments based on the acquired expertise (Figure 1). The ontology that we aim to generate with the framework we have developed is primarily based on, but not limited to, chemical structure. We operate on the premise that chemical class sub-specialization is enabled through chemical structure extension. We claim that for each class, a collection of functional groups exist that are necessary for a molecule to be considered a member of this class. This forms the basis for the self-organization of our automatically generated chemical ontologies.

### Automated Derivation of Formal Chemical Class Definitions

Given a set of chemical entities known to be members of a given chemical class, our framework is capable of abstracting and generalizing a set of consensus molecular sub-graphs and features that are present in all representative members of this class (see Methods). In identifying such features, we consider not only the chemical graph that is formed by the overlap of several smallest class representatives, but also the chemical sub-graphs formed by the fragmentation of class members, and atomic features, such as ring membership, aromaticity, and connectivity. Therefore, we generate a set of both, canonicalized SMILES [20] and canonicalized SMARTS [21] patterns (see Methods) that are present in a given molecule. Canonicalization of SMILES and SMARTS patterns is important because the fragmentation of chemical structures and their generalized forms often yields chemical patterns that are equivalent, but can be written down as valid SMILES or SMARTS in multiple ways. Canonicalization of consensus patterns ensures that each pattern has exactly one definition, thus allowing for facile integration and comparison of consensus fingerprints from multiple class definition generation procedures.

**Figure 2:**
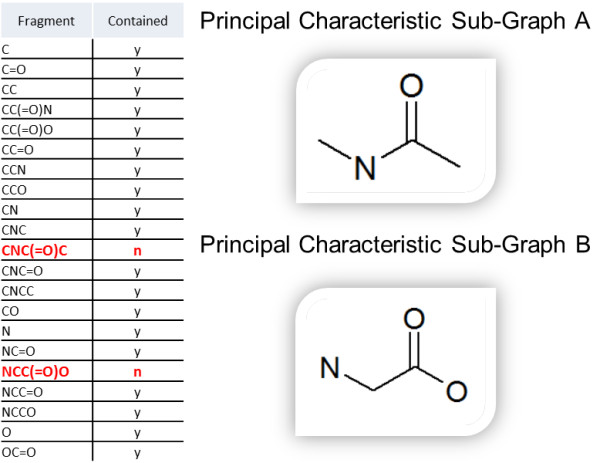
**Some of the consensus structural features identified for the class of peptides using the proposed methodology**. Note that in the two consensus fragments that are not contained in other consensus fragments, the basic peptide backbone is conserved, even for proline, a highly exotic amino acid, since 'N' in SMARTS does not necessarily mean an NH3 in practice.

The defining features, or chemical structural motifs, that are present in every member of a given class are deemed to be necessary for class membership and are termed consensus chemical features and their collection may be viewed to constitute a consensus chemical fingerprint for a given chemical class. The consensus chemical features that contain but are not contained in other consensus chemical features are termed by us *principal characteristic substructures*. These substructures can be used directly by curators for quick validation of the automatically generated class definition. For instance, for the ChEBI Peptide class (CHEBI:16670), one would expect the principal characteristic substructure to constitute the amino acid backbone (Figure 2), which is indeed the case.

In the process of determining principal characteristic substructures, we have identified certain classes for which our algorithm could not converge on an expected substructure set. Surprisingly, this was observed for carboxylic acid esters, where only the pattern associated with a carboxylic acid, but without extension to create an ester was found. Upon closer investigation, we have discovered that these difficulties in convergence were exclusively due to errors in the manual curation of input data. In the case of carboxylic acid esters, the failure of convergence was due to the inclusion of a molecule containing RC(= O)ONR pattern instead of the RC(= O)OCR pattern in the training set. Technically, carboxylic acid esters conform to a pattern RC(= O)OR', where both, R and R' are aryls or alkyls [22], meaning that the inclusion of the nitrogen-linked ester derivative was erroneous. This was a very fortunate development, since this identified one possible method to verify the consistency and correctness of human curators.

**Figure 3:**
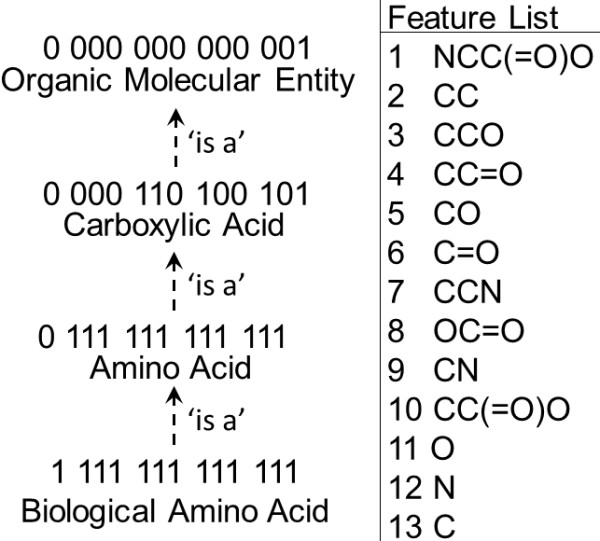
**Establishing a chemical class hierarchy through direct observation of consensus structural fingerprints is similar to logical hierarchy construction**. It is possible to infer parent/child relationships based solely on the consensus features of a class. Formal logic affords much more powerful expressions.

Our second premise is that for one class to be considered a child class of another chemical class, it has to contain all the consensus structural features of the parent class, and some additional features specific to it only (most likely, but not necessarily, principal characteristic substructures). Therefore, while the principal characteristic substructures will be useful in establishing the unique structural identity of a given class, the rest of consensus structural features are useful in establishing the class hierarchy (Figure 3). This basic premise applies to both, simple consensus structural fingerprints, and the formal logical definitions of chemical classes and forms the foundation of the self-organizing nature of the chemical ontology generated by our framework.

The next step in self-organizing chemical ontology generation is the formalization of chemical class definitions based on the consensus structural fingerprints. For this, we generate and maintain a single OWL ontology that is constantly updated with the unique consensus chemical substructures that are identified as a result of class analysis, using previously reported principles of unique, canonical, and invariable URIs for each functional group class [19]. The functional groups defined in this ontology are then used in converting the consensus structural fingerprints. For instance, a class of 'Organic Alcohols' is found to contain the consensus structural features expressed in SMARTS notation as [#6] (any carbon atom, aromatic or not), [#8] (any oxygen atom), [#6][#8] (carbon and oxygen in a single bond), and [#6][OX2H] (carbon connected to an OH group). This can be translated into the following equivalent class statement in OWL using the Manchester DL query syntax [23], which states that an instance of the class of organic alcohols is exactly defined by being an instance of a molecular entity and having at least one (keyword: some) instance of each of the four functional groups:

'Organic Alcohols'

EquivalentTo

'Molecular entity'

and 'has part' some '[#6]'

and 'has part' some '[#8]'

and 'has part' some '[#6][#8]'

and 'has part' some '[#6][OX2H]'

Clearly, this specification is human-readable, and can be further enriched by adding annotations on the functional groups involved, including graphical representations or textual descriptions for the various functional groups, in order to facilitate chemical class definition understanding.

Further, if one wishes to handle cases where more expressive logical statements are necessary, this is always an option. Consider, for example, the class of Glycols (also sometimes referred to as Diols), which require the presence of a minimum of two alcohol functional groups in a given molecule. Please note that we state that diols contain at least two alcohol functional groups, since in our approach, triols should be identified as simply a subclass of diols. The cardinality restriction to at least two alcohol functional groups allows for this. Thus, diols can be defined as follows. Please also note that cardinality is not currently screened for by the framework proposed here and all the cardinality restrictions in our automatically generated ontologies were added manually.

Diols

EquivalentTo

'Molecular entity'

and 'has part' some '[#6]'

and 'has part' some '[#8]'

and 'has part' some '[#6][#8]'

and 'has part' min 2 '[#6][OX2H]'

Attributes and molecular descriptors can also be handled with such definitions. For instance, we may find out that a given enzyme catalyzes the oxidation of organic alcohols that are no heavier than 500 Daltons. This mixing of chemical sub-graphs and physicochemical attributes is also possible, using the principles and concepts defined in the CHEMINF ontology [24].

'Reactive Diols'

EquivalentTo

'Molecular entity'

and 'has part' some '[#6]'

and 'has part' some '[#8]'

and 'has part' some '[#6][#8]'

and 'has part' min 2 '[#6][OX2H]'

and 'has attribute' some ('molecular weight' and 'has value' [= < 500] and 'has unit some 'Dalton')

Using the dataset of diols annotated with their computed molecular weight with the concepts from the CHEMINF ontology, we were able to correctly identify that all 14 diols in the ChEBI training set for this class were indeed 'Reactive Diols' as per the logical definition above.

### Self-Organizing Chemical Ontology

**Figure 4:**
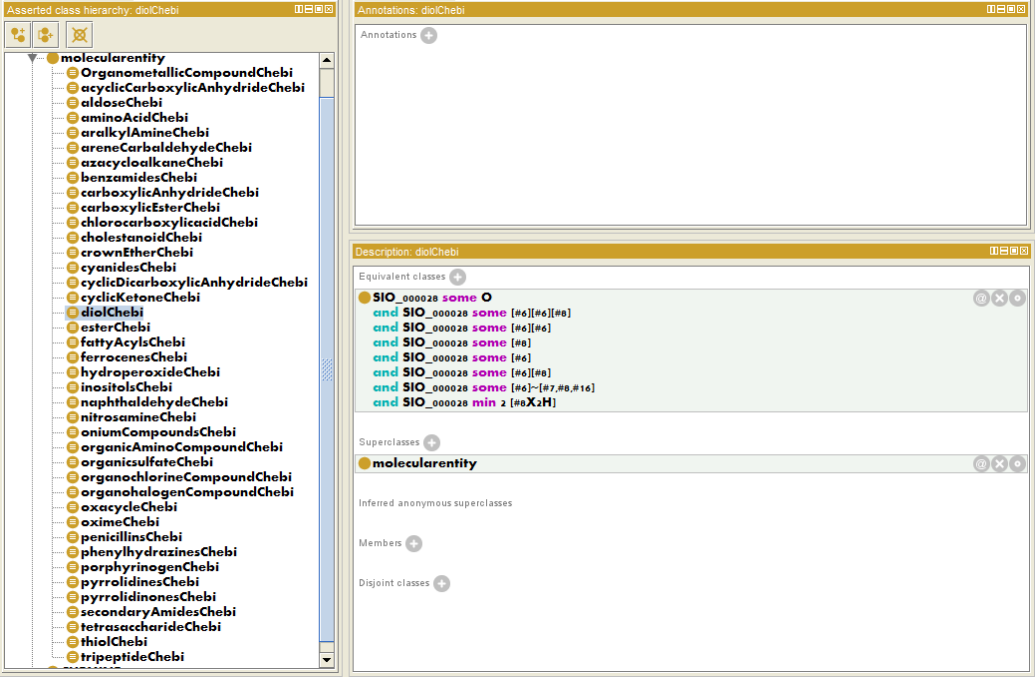
**An example of a 'flat' chemical ontology with chemical class definitions, within the Protégé 4.1 ontology editor**. No classification has been carried out.

**Figure 5:**
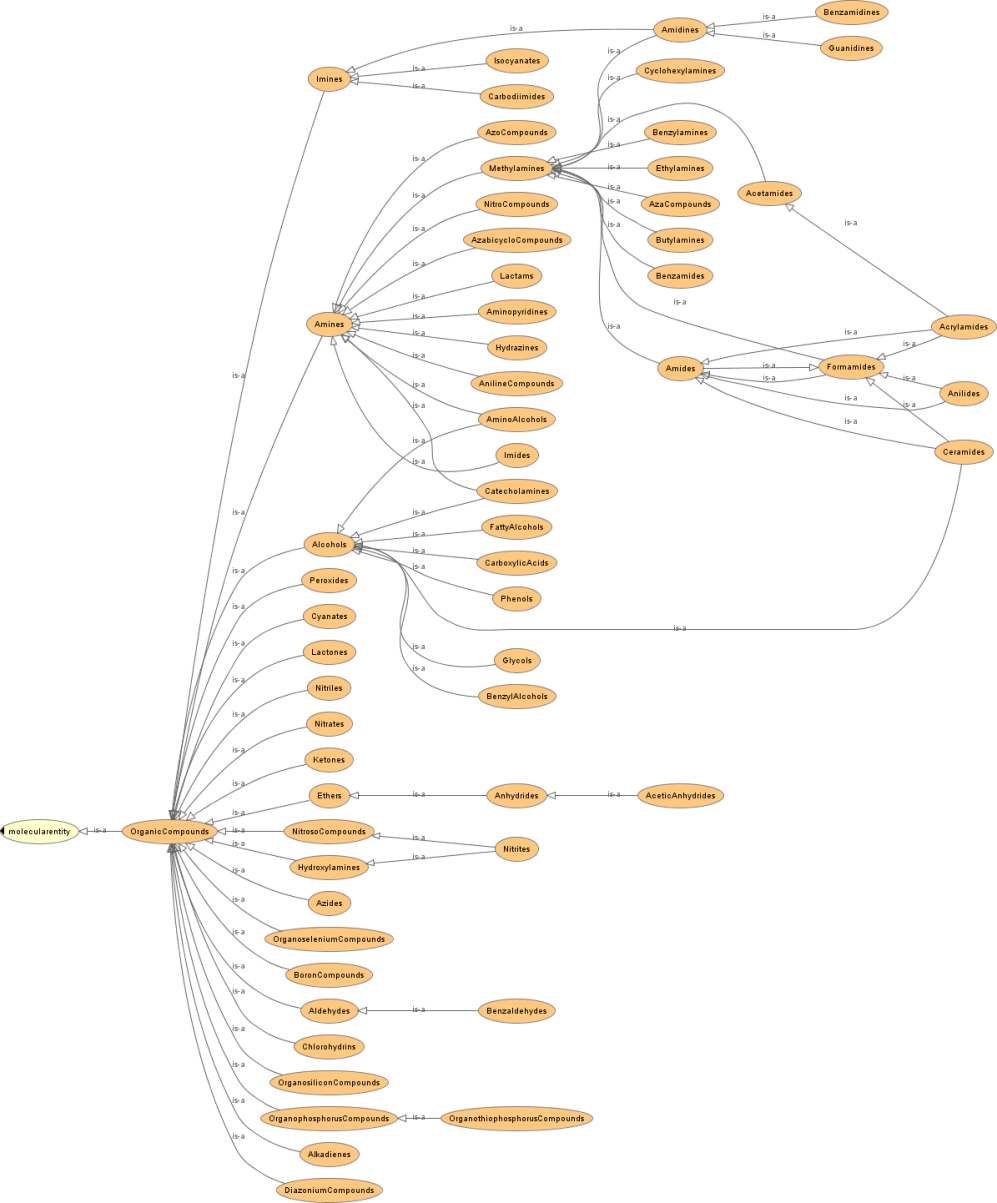
**MeSH chemical class hierarchy automatically inferred using the Pellet reasoner**. Please note that all 'is-a' relationships except those to 'molecularentity' are inferred automatically. Please note that all classes deal with organic chemicals.

**Figure 6:**
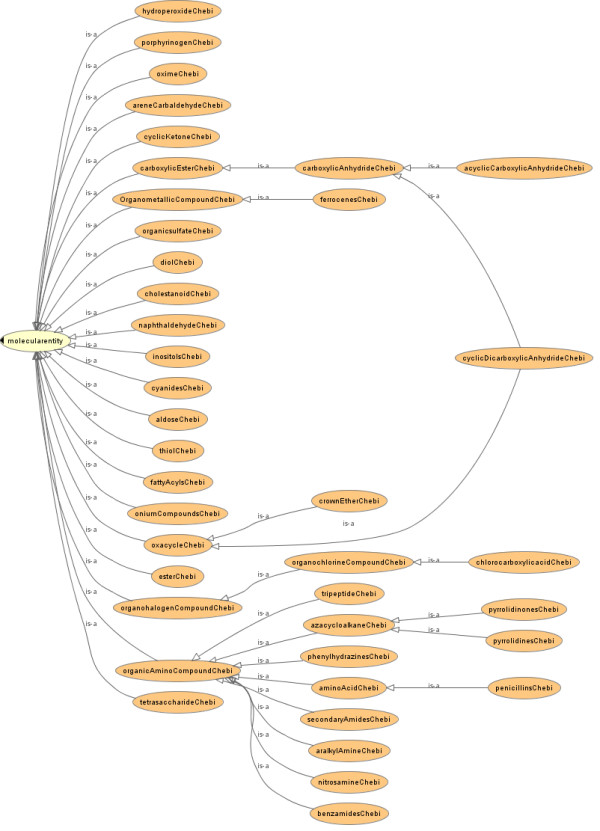
**ChEBI chemical class hierarchy automatically inferred using the Pellet reasoner**. Please note that all 'is-a' relationships except those to 'molecularentity' are inferred automatically. Please note that all classes deal with organic chemicals.

In order to test the integrative capacity and accuracy of our chemical classification framework, we have manually generated a dataset containing 40 ChEBI and 60 MeSH chemical classes, for a total of 100 classes with at least 7 and at most 20 representatives in each chemical class. To demonstrate the self-organizing chemical ontology creation from a specific source, we have created a stand-alone self-organizing ontology for each, the ChEBI and the MeSH data sets, containing the formal class definitions derived from consensus chemical fingerprints of each class (Figure 4). Upon application of the Pellet reasoner to classify these ontologies, we observed self-organization of the formally axiomatized chemical classes into chemical ontologies with a hierarchy (Figures 5 and 6). Each child-parent inference was screened by a curator to identify that the automatically generated ontologies were consistent for both datasets. In this screening, we have considered the structural viewpoint - that is, it was permissible for *ethylamines*, for example, to be classified as the child class of *methylamines*, since an ethylamine constitutes a structural extension of a methylamine, and because the training sets obtained for these classes (from MeSH in this case) included among methylamines chemical entities that were derivatized on the carbon atom that should have otherwise been terminal (a CH_3 _carbon). While we do not make any claims with respect to the validity of this approach, we note that this sub-classification inference in our work mirrors the manually assigned sub-classification in the MeSH hierarchy of organic chemicals.

**Figure 7:**
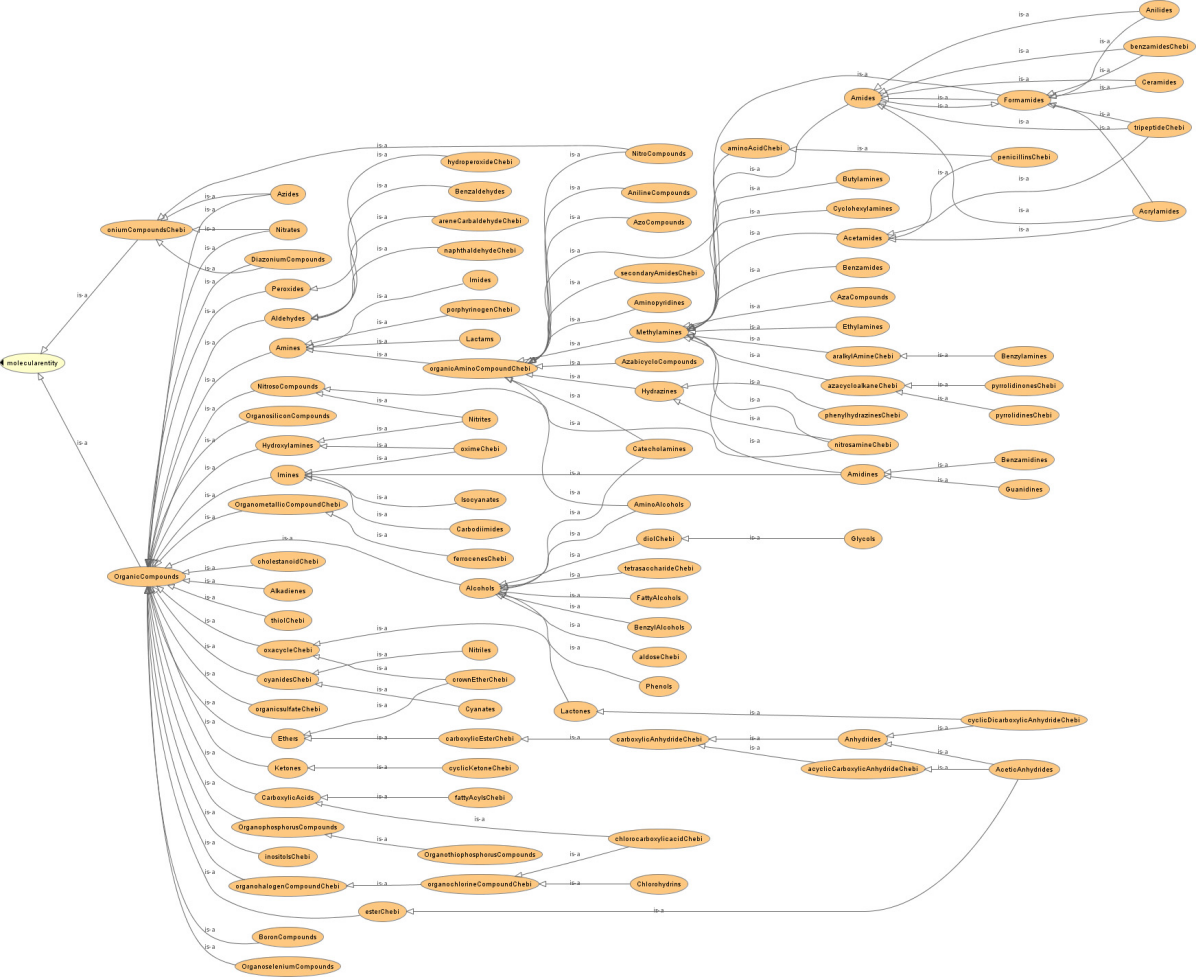
**The chemical class hierarchy inferred for an ontology that has resulted from the automated integration of the MeSH and ChEBI ontologies**. Please note that all classes deal with organic chemicals.

Because both ontologies were built using one central functional group ontology and the CHEMINF ontology for chemical descriptors, we were then able to trivially import the ChEBI ontology into the MeSH ontology within Protégé 4.1 [25] by specifying direct ChEBI ontology import within the MeSH ontology, and to apply the Pellet machine reasoner [26] to the resultant ontology to reconstitute a unified chemical ontology with 100 chemical classes (Figure 7). Thus, for the first time, we were able to automatically and trivially integrate and re-use the manual curation efforts from multiple data sources in order to construct a single seamless chemical ontology. Manual analysis of the class relationship inferences was carried out to ensure that the inferred chemical hierarchy was consistent and coherent from the viewpoint of chemical structure.

### Chemical Classification Accuracy

For a demonstration of chemical classification, we have randomly selected a set of 200 chemical entities and employed the integrated ontology in order to classify each individual compound. Because each chemical entity could be classified into multiple classes, the number of class membership inferences (452) exceeded the number of tested chemical entities. Within this dataset, 91% of chemical entities received inferences that exactly matched their annotation within the test set and additional 8.5% were matched to classes consistent with their test set-assigned class. For example, if a compound was a member of the alcohols in the test set, but actually contained two OH groups, this compound was correctly identified as belonging to diols or glycols, without the explicit specification of its membership among the alcohols, even though this membership is implied.

Overall, 92.7% of the entity inferences were correct (Table 1). Most of the observed errors were in the classes that necessitated the definition of negations. For example, a carboxylic acid is a structural derivative of an alcohol, but is strictly speaking not one. From a structural perspective, classification of a carboxylic acid as an alcohol is not incorrect. However, from a classical organic chemistry viewpoint, this is an incorrect classification, and has been noted as such in this work. In addition to this, we have observed that identifying incorrect inferences in some cases led to facile identification of adjustment of the training sets to automatically generate a more accurate chemical entity description or suggested manual refinements to definitions (e.g. glycols, diols, and alkadienes with cardinality restrictions). An additional table shows the classification results in more detail (see Additional file 1).

**Table 1:** Summary statistics for the results of automated classification of chemical entities in the test set

Tested Category	Total Inferences	Direct Inferences	Correct Inferences
Acetamides	13	4 (30.8)	13 (100.0)

Acetic Anhydrides	11	5 (45.5)	9 (81.8)

Acrylamides	20	4 (20.0)	19 (95.0)

Alcohols	9	4 (44.4)	9 (100.0)

Aldehydes	6	2 (33.3)	6 (100.0)

Alkadienes	8	5 (62.5)	8 (100.0)

Amides	2	1 (50.0)	2 (100.0)

Amines	9	0 (0.0)	9 (100.0)

Amino Alcohols	7	3 (42.9)	7 (100.0)

Aminopyridines	12	3 (25.0)	10 (83.3)

Anhydrides	5	3 (60.0)	5 (100.0)

Aza Compounds	3	1 (33.3)	3 (100.0)

Benzaldehydes	11	4 (36.4)	11 (100.0)

Benzyl Alcohols	10	4 (40.0)	10 (100.0)

Benzylamines	10	4 (40.0)	10 (100.0)

Boron Compounds	8	5 (62.5)	7 (87.5)

Butylamines	11	5 (45.5)	11 (100.0)

Carbodiimides	2	1 (50.0)	2 (100.0)

Carboxylic Acids	9	0 (0.0)	4 (44.4)

Chlorohydrins	11	5 (45.5)	10 (90.9)

Cyanates	4	3 (75.0)	4 (100.0)

Cyclohexylamines	6	3 (50.0)	6 (100.0)

Diazonium Compounds	15	5 (33.3)	10 (66.7)

Ethers	6	5 (83.3)	6 (100.0)

Ethylamines	4	2 (50.0)	4 (100.0)

Fatty Alcohols	2	2 (100.0)	2 (100.0)

Formamides	7	5 (71.4)	7 (100.0)

Glycols	6	2 (33.3)	3 (50.0)

Guanidines	15	5 (33.3)	15 (100.0)

Hydrazines	11	3 (27.3)	11 (100.0)

Hydroxylamines	14	5 (35.7)	13 (92.9)

Imides	21	5 (23.8)	21 (100.0)

Imines	7	2 (28.6)	7 (100.0)

Isocyanates	10	5 (50.0)	7 (70.0)

Ketones	6	5 (83.3)	6 (100.0)

Lactams	17	4 (23.5)	17 (100.0)

Lactones	10	5 (50.0)	10 (100.0)

Methylamines	7	3 (42.9)	7 (100.0)

Nitrates	6	2 (33.3)	6 (100.0)

Nitriles	8	5 (62.5)	8 (100.0)

Nitrites	5	5 (100.0)	5 (100.0)

Nitro Compounds	22	5 (22.7)	17 (77.3)

Nitroso Compounds	13	4 (30.8)	11 (84.6)

Organic Compounds	2	1 (50.0)	2 (100.0)

Organophosphorus Compounds	18	5 (27.8)	16 (88.9)

Organoselenium Compounds	6	3 (50.0)	6 (100.0)

Organosilicon Compounds	6	5 (83.3)	6 (100.0)

Organothiophosphorus Compounds	8	5 (62.5)	8 (100.0)

Peroxides	5	5 (100.0)	5 (100.0)

Phenols	8	5 (62.5)	8 (100.0)

Total	452	182 (40.3)	419 (92.7)

The direct inferences are class inferences that are identical to the annotations in the test set. Correct inference counts include the direct inferences and inferences that were deemed correct by a curator. Please note that a lack of direct inferences does not reflect an error - merely the presence of another class whose definition was a closer match for a given molecule than its original class. More than one inference was possible for a given molecule. Percentages of total inferences for each class are given in brackets for each category.

The entire classification process for 200 compounds took 34 minutes and 10 seconds, running on a Core i7-870 machine with 6GB of RAM, executed serially, while the computational time to classify non-populated ontologies was negligible.

### Seamless Integration of Novel Chemical Classes: Enzyme Substrates

In order to demonstrate the flexibility and facile amenability of our ontology to the incorporation of novel chemical classes, and especially chemical classes of biological relevance, we have created a stand-alone class of chemical entities that are substrates to yeast alcohol dehydrogenase, using information in the BRENDA database [27]. Because this data was experimentally derived and the reactions indicated were confirmed to occur in nature, no human curation (beyond eliminating redundant entries) was necessary for the training set composed of 23 unique chemical entities. Upon the repetition of the exercise described above with this chemical class, we have, unsurprisingly, discovered that the resultant class of yeast alcohol dehydrogenase substrates was a subclass of alcohols, with the added requirement of possessing an oxygen atom connected to one hydrogen atom and a non-aromatic carbon atom.

## Discussion

Here, we have presented a radically streamlined framework and approach to chemical ontology construction based on explicit formal semantic specification of chemical classes and rooted in chemical graph analysis. By eliminating the manual assertion of ancestry relationships between the various chemical classes and instead emphasizing the adequate curation of the limited (as small as five entities) training set of the starting chemical class representatives and the automatically generated logical class definitions, we have greatly simplified the work of human curators, thus potentially improving their productivity and making the annotation and classification of the rapidly multiplying chemical information more manageable.

One may argue that curators may also easily define chemical patterns in order to define a given chemical class instead of starting with identifying a diverse set of compounds that would then be used to automatically derive the relevant patterns. While this is indeed true, automated consensus chemical structure identification has numerous benefits, including the exhaustive identification of primitive structural features that are useful in constructing class hierarchies, as well as in the inherently objective identification of the structural patterns of interest. Manual supplementation of class definitions is always possible, as we have shown with class customization, and is sometimes required, in cases where our algorithms cannot identify a consensus pattern despite the best efforts of the curators to include only valid structures in the training sets.

For example, classes that include much variability within the key structural elements may not be adequately characterized by our algorithm as it stands currently. For example, the IUPAC-recommended interpretation of the term 'ester' includes compounds that conform to C(= O)OC (i.e. carboxylic esters) and C(= S)OC patterns, among others [22]. If entities representing both of these patterns were grouped into a single training set, a single descriptive pattern would not be identified with our current algorithm, since there is variation on the central atom. In this case, a human curator's intervention may be necessary to include a pattern such as C(=[O,S])OC that describes both cases.

Although this is a difficult problem to address, it is not unavoidable with future improvements to the consensus chemical pattern identification algorithms. For example, had we not considered the atomic ring membership property in the generation of our consensus chemical patterns, we would not have been able to adequately model the class of cyclic organic compounds. One approach around this would be to manually define a feature [#6;R] and screen for this feature to identify whether it is a consensus chemical feature for a given class. Another, much more labour-intensive approach would be to identify the cyclic organic compound sub-classes and explicitly enumerate them in the definition of this class, as follows.

'Cyclic Organic Compounds'

EquivalentTo ' Molecular Entity' and 'has part' some Cycle

'Three-Membered Cycle' subClassOf Cycle

'Five-Membered Cycle' subClassOf Cycle

Fortunately, our algorithm currently identifies and screens for the presence of a number of ring features and abstracts chemical structures so as to include considerations for atoms' hydrogenation state (such as OH versus O, CH3 versus other forms of carbon atom), ring membership, aromaticity, general type (such as [#6] is any carbon atom), as well as combinations of these attributes. Thus, the feature [#6;R] is automatically identified by our algorithm by examining the chemical entities in the training set. In addition to this, we screen for 396 pre-defined patterns designed to capture the nuanced chemical class definitions, such as the general ester feature discussed above, and we screen each class with all the previously-generated consensus patterns in the other class definitions. This allows us to handle the definition of many more classes than just those with automatically generated consensus substructures, including, for example, bicyclic molecules, which are composed of two fused rings and are characterized, among other automatically identified features, with the manually pre-defined [R2] SMARTS pattern, which defines any atom contained within two rings simultaneously.

While some cases can be handled automatically in principle, for some classes of compounds such as Nanotubes (CHEBI:50796) or Polycyclic Cages (CHEBI:33640), manual curation is unavoidable, as we do not yet have the capacity to adequately characterize the requisite consensus chemical feature information in terms of SMILES, SMARTS, or properties, but only some very complex logical expressions or expressions in some molecular query languages. This may potentially be handled automatically in the future by screening for the requisite higher-order chemical graph features and patterns using seamlessly integrated semantic web services with the SADI framework [28-30], but is not handled by us at present.

Another aspect that we would like to address in the future is the automated identification of consensus chemical feature values in order to investigate the handling of more biologically relevant chemical classes, as demonstrated for the 'Reactive Diols' class. While we have already demonstrated the learning and formal logical encoding of biological activity and toxicity decision trees, we have not yet connected that effort to structure-based classification. In addition to this, classes of biologically active compounds are rarely as well defined as they are for the alcohol dehydrogenase substrates. For instance, structurally distinct compounds may sometimes engage in a number of productive binding or interaction modes with the same enzyme. While such annotation is not our primary focus, we claim that each individual binding mode should be amenable to topology-based and consensus feature-based logical characterization as a sub-class of the broader enzyme substrate class. Failing that, as we have demonstrated, the existing chemical graph-based class definitions can always be easily augmented with various chemical property and geometric characterization restrictions manually.

Unfortunately, certain aspects of chemical class definition formulation are difficult to address in principle. Of particular concern is the negation of chemical statements in some class definitions. For instance, some people may argue that fatty acids constitute a class of compounds that should not contain any rings, among other features. This negative relationship is rather difficult to capture with the proposed methodology, and in fact, the majority of cases where classification of small molecules was incorrect were due to the lack of automatically generated negations in the class definitions. To name just one example of the consequences of this behaviour, due to the lack of negation detection, esters are inferred to be a subclass of ethers. While it is possible to correct this manually by including statements regarding the necessity of the absence of certain structural features in the class definition for ethers, automatic identification of such structural features is still challenging. The difficulty here is not posed so much by the identification of the features that are consistently absent from every member of a given class, as it is by the identification of which of the numerous features identified as absent from every member of a class should truly be absent. Of course, given a diverse enough training set for a given class, many of the chemical features would be eliminated from the list of forbidden features. However, one may never be entirely certain that the groups still on the list should indeed be forbidden in a given class, until all the possible class members have been included in the training set, thus defeating the purpose of automated classification.

The final aspect where human curation still has an edge is human-created, arbitrary or otherwise necessarily diverse groupings, such as is the case for vitamins and many natural products, or terms that refer to broad functional roles rather than chemical structure. Certainly, this is still very much a domain of subjective human classification where humans are irreplaceable, and will likely never be wholly replaceable by automated and objective chemical classification approaches. Thus, human involvement is necessary in our proposed mode of chemical ontology construction and refinement, but it is also more subtle, nuanced, and much more productive than it has ever been in any of the on-going chemical ontology construction efforts.

Having discussed the limitations and caveats of the proposed framework as it currently stands, let us examine more closely what it enables. By providing chemical class definitions in a logical and objectively consistent manner, we have created a means by which chemical hierarchies can be seamlessly integrated and new, previously uncharacterized classes can be added without necessitating a manual review of the whole ontology structure. The process of annotation of chemical entities has been greatly simplified and reduced to merely chemically fingerprinting a molecule and importing its CHESS-encoded fingerprint for facile automated classification with machine reasoning agents, all of which can be done on-the-fly as the chemical entities are loaded into a given ontology.

Finally, there is no further reason for compartmentalization and erection of artificial barriers between the ontologies derived by distinct chemical classification efforts. In principle, all chemical hierarchies can be fused into a single, consistent, and self-organized ontology, where chemical class equivalences and relationships are automatically inferred and reconciled. We have demonstrated this with the automatic integration of chemical classifications derived from multiple, independently-curated data sets, one using the MeSH-annotated PubChem molecules, and one that relied on ChEBI small molecule annotations. Previously, classification efforts in either manually created hierarchy stressed the manual establishment of explicit parent/child relationships between the various chemical classes, as well as potentially formulating a short description regarding what exactly went into deciding whether certain compounds should be annotated as members of a given class. In this regard, ChEBI is, beyond comparison, superior to the MeSH classification, because unlike MeSH it includes a programmatically examinable, formal ontology of chemical classes that can be queried for small molecule class membership. In addition to this, the ChEBI ontology is understandably includes many more terms and specific classes of biological interest.

Unfortunately, neither of the two chemical ontologies currently has means for facile integration with each other, nor facile class extension and inclusion of previously uncharacterized chemical classes. For both ontologies, the precise location of novel class addition is performed using subjective human assessment and examination of the possible child and parent classes. When a decision is made for the location of the class in the context of the chemical hierarchy, no formal justification is provided for the decision, meaning that the location of the class is a subject to errors and interpretation. On the other hand, in the formally specified ontologies that we generate by screening the data that is already available with PubChem and ChEBI, every subclass inference is explainable logically and amenable to facile correction through corrections in formal chemical class definitions rather than an unexplainable reshuffling of the class relationships in the current ontologies. Furthermore, the effort of adding a novel class into the ontology is limited to defining the class itself, rather than analyzing all the other existing classes for their potential role as parent classes or sub-classes of a given novel class. This is the principal reason for the ease of the integration of the MeSH and ChEBI ontologies. To summarize, the principal benefits of our proposed approach are i) the ease of chemical class integration into existing hierarchies, ii) facilitated chemical class definition and characterization, and iii) logically explainable and therefore easily correctable chemical class hierarchy assignments. Furthermore, to generate the hierarchies presented in this work, we have essentially re-used the previous work done by annotators on the ChEBI and MeSH projects already, with slight screening modifications of the existing data sets. This gives hope that the existing chemical hierarchies can be somewhat easily converted into their logically encoded forms.

The automatically generated ontology that we present here also removes much subjectivity and ambiguity from chemical class assignments and annotation of individual small molecules. It is no longer necessary to manually adjust the annotation of every small molecule that needs to be classified. This is of critical importance as the ChEBI effort transitions to the classifications of hundreds of thousands compounds present in the ChEMBL database, and the PubChem database experiences constant exponential growth of millions of new, partially characterized or completely uncharacterized compounds. The classification procedure for the novel small molecules presented here is highly accurate, and when each class is screened using a testing set, classification inconsistencies are highly visible and easily correctable. For example, the classes of alkadienes and diols necessitated cardinality restrictions in their formal definition to account for the fact that these classes required a given molecule to contain two alkene or two alcohol groups, respectively. Upon screening these classes with a test set of known positives and known negatives, the misclassification of known negative compounds containing only one of these groups has alerted us to the omission of cardinality restriction in the definition of these classes. While we anticipate adding automatic cardinality restriction identification in future work, this feature is currently not implemented and appropriate class definitions had to be manually corrected. Thus, awareness of the formal definition of these classes has alerted us to an omission in our work. On the other hand, there is no capacity or provision for explaining chemical classifications and annotations in the major chemical ontology building efforts, which means that any errors in chemical entity annotation assignment have to be manually identified by the users of these hierarchies, discussed by curators, and then manually corrected, in a further time-consuming endeavour. We shall reiterate: in the proposed framework, only class definitions need to ever be adjusted, while chemical entity classification is automatically inferred, and can be generated at will, for any arbitrary compound, and the chemical hierarchy is dynamically reconstructed.

We must note that the high degree of accuracy of automated small molecule classification is not surprising since we were operating on structurally-derived classes and we have performed a nearly exhaustive characterization of chemical structure to identify unambiguous, deterministic class definitions. This is in sharp contrast with the inherently probabilistic, 'black box' training approaches based on artificial neural networks or support vector machines, where features of chemical entities in a given training set are incorporated into (often difficult to interpret) mathematical expressions which are expected to be correct only a fraction of time, and due to the statistical nature of these approaches may involve chemical features that may have little to do with the proper classification of a given compound into a given class, in reality. Our task is very much simplified, as we do not concern ourselves with chemical features that only appear in a fraction of compounds within a given class, but rather admit to class definition only the consensus features.

## Conclusions

**Figure 8:**
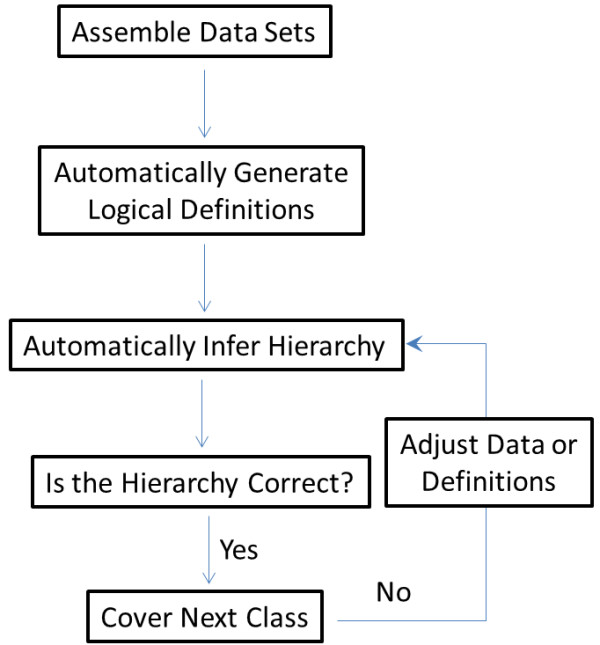
**A novel approach to chemical hierarchy construction, maintaining human involvement and supplemented with automated cheminformatics algorithms developed in this work**.

Based on our own experiences in constructing the chemical hierarchies presented here, we envision a new iterative approach to chemical ontology development (Figure 8). In this scheme, curators are primarily concerned with the assimilation of a representative set of chemical entities for each chemical class based on whether the desired principal chemical substructures for a given class have been identified. Manual adjustment of some class definitions and adjustment of other definitions to explicitly include disparate classes that may contain a set of well-defined, but disparate and non-overlapping sub-classes may also be performed. However, explicit specification of class membership or chemical ontology hierarchy is possible but not recommended in this approach to maintain the self-organizing and objective nature of the chemical hierarchy.

With the rising of systems sciences to prominence, we believe that chemical annotation and classification frameworks, such as the one presented here, shall increasingly rise in prominence and importance. The already impressive collection of chemical data on the Semantic Web and its fruitful applications in chemical research suggest that semantically enabled chemical classification systems are of immediate applicability and importance. Furthermore, the ability to formally specify and exchange chemical class definitions can open new doors to collaboration in chemical and biological research, driving new innovations and discoveries.

## Methods

### Training and Testing Data Sets

For our proof of principle work, we have identified 60 MeSH classes and 40 ChEBI classes that were subjectively identified as potentially related by a curator and which would thus be expected to either have equivalent logical definitions or identified as sub- or super-classes of each other. For MeSH-defined chemical classes, we have used PubChem to identify all compounds previously annotated with a given MeSH chemical class term. The resultant set of chemical structures was carefully manually screened by a curator to assure that the MeSH term has been correctly assigned in the training data and that the representative class instance set adequately covers the full spectrum of compounds included in the class. For ChEBI classes, we have used the ChEBI API to select only the high-confidence (three-star in the ChEBI star system) annotated chemical entities that were identified as instances of a given ChEBI chemical class. In order to assure complete adherence to the ChEBI class definition in the class instances, a curator was assigned to manually screen these instances with the same criteria as those for the MeSH data set. This resulted in 766 chemical entities manually screened for the MeSH data set and 606 chemical entities manually screened for the ChEBI data set. The screening process involved not only the querying of the appropriate databases for members of a given class, but also consultation with the existing literature on the definition of each class, as class definitions in ChEBI, and especially in MeSH were often lacking in detail. The training data sets are available from the project website [31] as well as attached with this paper (see Additional file 2).

### Identification of Consensus Molecular Fragments and Patterns

For the identification of consensus chemical substructures, structural patterns, and molecular skeletons, we have created custom software that relied upon the Chemistry Development Kit version 1.2.4 [32] and OpenBabel version 2.2.3 [33]. The software we have created is freely accessible upon request (see the project website [31]). For each class, the set of representative chemical entities is partitioned into a training set which contains at least five least complex representatives (in terms of the number of atoms and bonds) of the investigated class (manually assuring a complete coverage of the class), and a test set which is used to assess the accuracy of formal class definition. The assumption that allowed selecting the least complex class representatives for training is that the smallest class members have to contain all the consensus structural patterns that the rest of the class contains. This allows us to save on computational time.

A chemical substructure, pattern, or molecular skeleton is considered to be a consensus one for a given class if it is found to be present in all members of the training set. In order to identify chemical features to consider as candidates for consensus features, we employ a hybrid approach involving limited molecular fragmentation, subgraph isomorphism detection, predefined chemical feature detection, and the fragmentation of chemical entities with atoms that are annotated for full hydrogenation state (indicating a terminal atom in a chain, e.g. CH3 or OH), ring membership, aromaticity, general atom type, as well as binary combinations of these attributes including aromatic and fully hydrogenated atoms, ring and fully hydrogenated atoms, and general atom types and fully hydrogenated atoms.

To identify smaller consensus substructures, we have carried out only a limited molecular fragmentation since we were limited by the apparent and highly perceptible combinatorial explosion of possible fragmentation choices on large and ring-containing molecules. We have therefore limited the fragmentation to exhaustively consider all the possible fragmentation patterns resulting from cutting all possible combinations of up to four bonds in every molecule. If the fragmented compound was the native SMILES-encoded structure, each fragment was then canonicalized using OpenBabel and only the unique fragments were collected. For SMARTS-transformed compounds, the procedure was somewhat more involved (see below).

To identify principal characteristic substructures for each class, we have employed Chemistry Development Kit in order to compute the maximal common substructures in binary combinations of the five smallest class members. Again, the selection of a limited number of the smallest class members has been made in order to eliminate unnecessary computational expense. The principal characteristic substructure for a given class has to be present in every member of a given class, including the smallest members. This also means that one class member in the curated set should be selected such that it contains the smallest set of features while still qualifying as a class member. For example for the class of alcohols, this could be methanol.

The resultant collection of maximal common substructures was then used to derive the maximal common substructures of the maximal common substructures. That is, even if the smallest class members did not contain the one smallest member that is still representative of a given class, this procedure would have identified the principal characteristic substructure for a given class. Consider, for example, the class of Benzenes, which is composed of benzene and its derivatives. The principal characteristic substructure is the benzene ring. However, if we included among the smallest substructures only three different products of addition to the benzene ring, the ring itself would have been still identified as a result of this procedure. We accounted for all three smallest structures involving consensus features (such as if all were aniline-based) by also fragmenting the maximal common substructure thus identified using the fragmentation approach described above.

Finally, each member of the training set was screened for the presence of each feature identified using fragmentation and maximal common substructure analysis, along with 396 manually pre-defined features, obtained from a number of collections. The positive responses were tallied and the fraction of compounds that contained every given substructure or structural pattern was then computed. If this fraction was below 1.0 for a given fragment or pattern, it was discarded from the consensus fragment list. If, on the other hand, this fraction was 1.0, a given fragment or pattern was identified as a consensus structural pattern or fragment.

### SMILES and SMARTS Pattern Canonicalization

Canonicalization is important since, among other things, it helps prevent repetition and unnecessary screening of the chemical entities for features multiple times. The SMILES patterns obtained using the above approaches were canonicalized using the OpenBabel software. For SMARTS patterns, the situation was complicated by the fact that, to the best of the authors' knowledge, there is no single piece of software that can canonicalize SMARTS patterns. To accomplish this, we have used the fact that atomic mass has a bearing on the ordering of atoms within a canonical SMILES representation, as generated in OpenBabel. Thus, we have represented each atomic feature as a separate isotope type (e.g. a ring carbon is 14C, while an aromatic carbon is 15C) and used such SMILES strings in order to determine a canonical atom ordering using the normal SMILES canonicalization procedure in OpenBabel. After obtaining the canonical isotopic SMILES string for a given fragment, we converted it using a lookup table into a SMARTS pattern specification. This gave us a robust canonical form for every SMARTS pattern we generated.

### Chemical Class Definitions Encoding in OWL

In order to translate the consensus chemical fingerprints to OWL-based axioms, we have used the OWL Java API, version 3.2.3. We have set up a central ontology of functional groups and structural features, which is referenced by every ontology that contains chemical class definitions generated using the proposed methodology. This allows for facile chemical hierarchy integration from multiple distinct efforts. In the functional group ontology, all the functional groups are merely declared with a canonical URI, which is generated using reproducible SHA1 40-character hashes of the canonical functional group SMILES or SMARTS pattern. This ontology contains no hierarchy, but only the instances of functional groups and their labels (SMILES or SMARTS).

The ontologies containing chemical class definitions are generated by iterating over every consensus feature and adding class restrictions to the definition, through the 'has attribute' object property defined in Semanticscience Integrated Ontology, and using the 'some values from' restriction on this property. Cardinality restrictions were added manually to the existing ontologies using the Protégé 4.1 ontology editor, only for a very limited set of classes that required these restrictions.

### Chemical Classification Accuracy Evaluation

The accuracy of chemical class hierarchy was assessed by applying the Pellet reasoned, version 2.2, to each generated ontology, and then recording all the pairwise child-parent relationships within the resultant automatically generated hierarchies. The relationships that conformed to the hierarchies from which they were derived, as well as relationships that were acceptable according to the judgement of a curator were tallied up, and this score divided by the total number of the inferred relationships was reported as classification accuracy.

In order to classify chemical compounds using the formally axiomatized chemical class definitions, we represented 200 randomly selected chemical compounds from the MeSH data set (previously removed from training) using Resource Description Framework-based CHESS formalism, with the Jena Java API version 2.6.2 [34]. Each compound was screened to detect the presence of each of the chemical features defined in the central functional group ontology. Unique occurrences of functional groups within each chemical entity were individually instantiated and mereological relationships between these functional group instances and their containing chemical entity instance were defined. Each instantiated entity was identified explicitly as distinct to allow classification into cardinality restriction-containing classes. For the chemical entities that were used to demonstrate chemical class definition extension, we have also generated CHESS-encoded molecular weight descriptors. After this instance data in the RDF/XML form was imported into the ontology received from the integration of the MeSH- and ChEBI-derived ontologies, the Pellet reasoner was used again to realize the ontology with instances. Each chemical class inference was then manually screened by a curator and scored for exact matches to the test set data, correct classifications, and incorrect classifications.

## Availability and requirements

The source code for our generator, the input data and the generated ontologies for this study are available from the project website [31].

## Authors' contributions

LLC wrote the manuscript, ran all experiments, generated training data, and wrote all the software for this work. JH provided some data from ChEBI and provided helpful discussions of the work. CS and MD helped direct this work. All authors have read and approved the final manuscript.

## Supplementary Material

Additional file 1 **Detailed analysis of results of automated chemical annotation by human curators**. This file contains the results of automated chemical entity classification as well as the assessment of these classifications by a human curator as correct and direct (inferred classification is identical to training set classification). Comments on decisions to view a particular classification as erroneous or correct are also included. Click here for file

Additional file 2 **Chemical entities to train and assess definitions for each class**. This file contains the complete collection of chemical entities collected by human curators to compute chemical class definitions as described (see Methods). The names for each class as used in this work are also reported, along with the class ID for the ChEBI classes. Click here for file
